# Generation of Supra-Wavelength Grooves in Femtosecond Laser Surface Structuring of Silicon

**DOI:** 10.3390/nano11010174

**Published:** 2021-01-12

**Authors:** Jijil JJ Nivas, Salvatore Amoruso

**Affiliations:** 1 Dipartimento di Fisica “Ettore Pancini”, Università di Napoli Federico II, Complesso Universitario di Monte S. Angelo, Via Cintia, I-80126 Napoli, Italy; 2 Consiglio Nazionale delle Ricerche-SuPerconducting and Other INnovative Materials and Devices Institute (CNR-SPIN), UOS Napoli, Complesso Universitario di Monte S. Angelo, Via Cintia, I-80126 Napoli, Italy

**Keywords:** laser-induced periodic surface structures with supra-wavelength period, femtosecond laser surface processing, laser surface texturing, silicon

## Abstract

Extensive research work has been carried out on the generation and application of laser-induced periodic surface structures (LIPSS). LIPSS with a sub-wavelength period generated by femtosecond laser irradiation, generally indicated as ripples, have been extensively investigated. Instead, the other ordered surface structures characterized by a supra-wavelength period, indicated as grooves, have been much less studied. Grooves typically form at larger irradiance levels or for higher number of laser pulses. Here, we report a comprehensive overview of recent investigations on the supra-wavelength grooves formed on crystalline silicon irradiated by femtosecond laser pulses. The authors’ recent experimental work is mainly addressed giving an explicit picture of the grooves generation process, namely illustrating the influence of the various experimental parameters, including, e.g., polarization, wavelength, fluence and repetition rate of the laser beam as well as number of laser pulses hitting the surface of the material. The effect of irradiation of a static or moving target and of the environmental conditions (e.g., vacuum or air ambient) will also be discussed. Finally, possible mechanisms envisaged to explain grooves formation and still open issues are briefly discussed.

## 1. Introduction

The observation that regular structures form on the surface of a semiconductor target irradiated by a ruby laser beam was firstly reported by Birnbaum very early after the invention of the laser [1]. In the following two decades, a number of studies followed evidencing a rather universal nature of the phenomenon of laser-induced periodic surface structures (LIPSS) formation on a variety of materials irradiated by nanosecond (ns) and picosecond (ps), linearly polarized, laser pulses [2,3,4,5]. The experimental observations were rationalized by relating the generation of the surface features to scattered waves induced by surface roughness or defects that interfere with the laser field producing a spatial modulation of the absorbed energy with a typical periodicity of the order of the laser wavelength, which is eventually engraved on the target surface modifying its state or topography [2,3,6,7]. With the advent of femtosecond (fs) laser sources, the experimental observations of other surface structures with a period much smaller than the laser wavelength [8,9,10] triggered a renovated interest on the topic. The subsequent flourishing experimental and theoretical investigations led to the development of a realm of applications related to the variations of the surface responses due to the LIPSS formation, making such a subject a timeless field of research [8,9,10,11]. A fascinating characteristic of the LIPSS is the possibility to texture large surface areas by appropriate scanning of laser beam or sample [11,12,13], which is of paramount importance for applications. Nowadays, laser generated surface texturing allows creating, for example, surfaces with [10,11,12,14,15,16,17,18]: (i) novel optical properties such as “black silicon”, “black metals”, or with structural colors; (ii) peculiar wetting properties ranging from super-hydrophilic to super-hydrophobic; (iii) novel tribological responses; (iv) features that mimic natural species and their distinctive functionalities; and(v) particular qualities with applications in photonics, energetics, data storage, sensing, biology, and so forth.

LIPSS with a sub-wavelength period are generally indicated as "ripples" and classified into high spatial frequency LIPSS (HSFL) and low spatial frequency LIPSS (LSFL) depending on the characteristic period observed for irradiation at normal incidence [10,18,19]. The former structures are generated only by fs and ps laser pulses and have characteristic periods *Λ* much smaller than the laser wavelength *λ* (Λ < *λ*/2), whereas the latter show a periodicity smaller but comparable to the laser wavelength (*λ*/2 ≤ Λ ≤ *λ*) and constitute fs-laser-generated counterpart of the classical LIPSS observed earlier. Ripples have been extensively investigated and the various aspects related to their formation have been illustrated in a number of reviews [10,11,12,14,15,16,17,18,19,20]. In particular, very recently Bonse and Gräf have reviewed the current theories of LIPSS formation addressing the several approaches that have been used to elucidate the various aspects of their formation [19].

Notwithstanding the wide attention given to the subject, the formation of ordered surface structures characterized by a supra-wavelength period and not yet included in the previous classification, generally indicated as "grooves", has been much less investigated. Grooves are preferentially aligned along a direction parallel to the laser polarization and their generation usually occurs at larger fluence values and for higher number of laser pulses with respect to ripples [10,21,22,23]. Grooves formation has been first reported in semiconductors (Si, InP, etc.) [21,22,23,24] and successively observed also in dielectrics (e.g., SiO_2_) [25,26] and in various metals and alloys (e.g., Ni, Ti, steel, etc.) [27,28,29]. However, grooves formation mechanisms and the dependence of their characteristic features on the various experimental parameters (e.g., laser wavelength, environment, etc.) has yet to be fully clarified. For example, differently from ripples, the periodicity of the grooves increases as a function of both laser fluence and pulse number [25,26,27,28,29,30,31,32,33,34]. These observations, in turn, suggest that the mechanisms leading to supra-wavelength grooves generation might involve processes different from those leading to ripples formation that still deserve careful investigations. Among the various mechanisms involved in the creation of the supra-wavelength periodic structures, spatial energy redistribution due to the interaction of the incoming laser beam with surface wave that might be induced by ripples and hydrodynamic processes related to wave phenomena have been considered [20,25,26,27,30,34]. Moreover, some experimental findings on grooves formation on a silicon target also suggest a possible effect due to nanoparticles back-deposited on the sample surface during the laser irradiation process [16,26,27,28].

Despite the limited number of systematic investigations on grooves, it is worth noticing that their orientation parallel to laser polarization has enabled a more direct visualization of the polarization state of the intense fs laser beams of structured light [32,35,36,37,38,39,40], whereas their larger periodicity and tunability range might also be of interest to further explore possible applications of fs laser-induced surface structures.

Here we report an overview on the recent investigations carried out on the supra-wavelength grooves formed on a crystalline silicon target by irradiation of a target surface with fs laser pulses, with emphasis on the results obtained by the authors. Wherever possible, the LSFL generated in the same experimental conditions will be considered for comparison. Silicon is chosen for its wide technological importance, well-known physical properties, and capability of forming a variety of surface structures. Recent experimental findings will be illustrated aiming at providing a rather thorough picture of the various features of grooves generated on crystalline silicon. In particular, the effects of various experimental parameters, including, e.g., polarization, wavelength, fluence, and repetition rate of the laser beam as well as number of laser pulses hitting the surface of the material will be illustrated. Moreover, the influence of irradiation on a static or moving sample as well as the possible impact of the environmental conditions (e.g., vacuum or air ambient) will be also discussed. Finally, possible mechanisms discussed in the current literature envisaged to explain grooves formation and their agreement with the experimental results as well as still open issues will be briefly reviewed. The paper is organized as follows: Section 2 briefly reports the general characteristics of an experimental setup used for direct fs laser surface structuring. In Section 3, the structures generated by a Gaussian beam are illustrated evidencing the conditions for grooves formation, whereas Section 4 is devoted to the presentation of spatially variant grooves produced by fs complex light beams. Then, in Section 5 the influence of various experimental parameters (e.g., laser fluence and pulse number, wavelength, etc.) on the grooves features is addressed. In Section 6, the hindering of groves formation observed for some specific processing conditions (e.g., in vacuum, for a circularly polarized beam and at high repetition rate) is discussed. Finally, Section 7 reports a brief summary of the current theoretical description of the possible mechanisms underlying the grooves formation.

## 2. Surface Structuring by Direct fs Laser Pulse Irradiation

**Figure 1:**
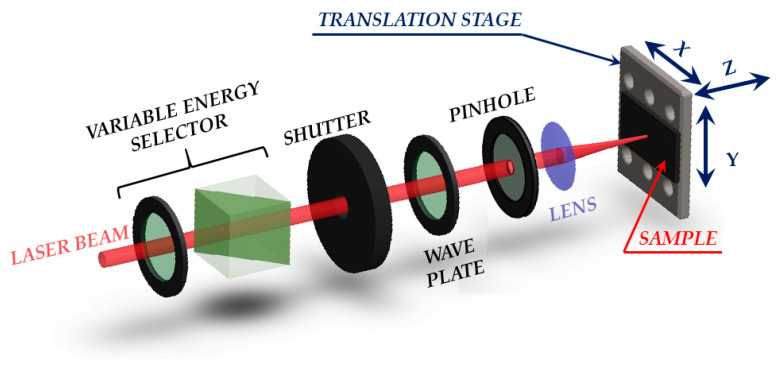
Sketch of a typical setup used in direct femtosecond (fs) laser surface structuring. The variable energy selector is formed by a half waveplate and a polarizer.

In recent years, direct fs laser surface structuring stands out as a very active method in surface structuring thanks to its simple experimental implementation, great versatility in texturing any kind of material, and large capability in producing many different surface structures suitable for a wide series of applications. As an example, Figure 1 reports a sketch of a typical setup used in direct fs laser surface structuring. A variable energy selector, formed by a half wave plate and a polarizer or an attenuator, is generally exploited to control the laser pulse energy. The number of laser pulses reaching the target is selected by means of an electromechanical shutter. A wave plate can be used to control the polarization of the beam impinging on the target, whereas a pinhole can select the most intense part of the beam. Finally, the laser beam is focused by a lens on the surface of the sample target that is mounted on a computer-controlled translation stage. When the translation stage sits still in predefined positions, this simple system allows producing series of single spots with a given number of laser pulses *N* defined by the aperture time of the shutter and the laser repetition rate *f_L_*. Instead, if the shutter is left open and the sample translated along the X- or Y-axis, a line can be formed by raster scanning. Finally, appropriate sample movement along the X- and Y-axis may lead to structuring over a larger bidimensional area. The Z-axis adjustment can be used to fix the position of the target with respect to the focusing lens.

In direct laser structuring laser beams with a Gaussian spatial intensity profile are typically used. In most cases a pinhole is inserted in the beam path to remove any possible distortion at larger distances from the propagation axis. The pinhole aperture is selected in order to minimize any diffraction effect. However, the insertion of suitable beam converters in the beam path can also allow structuring with more complex beams, as will be for example discussed in the following Section 4. For the generation of grooves with periodicity at the microscale, weak focusing conditions of the laser beam are employed in order to allow the formation of several structures over the area of the shallow crater induced by laser irradiation.

The morphology of the generated structures is usually analyzed by means of optical microscopy (OM) and scanning electron microscopy (SEM), while topographic information is obtained by exploiting atomic force microscopy (AFM).

Crystal orientation of the sample target can influence the formation of fs LIPSS [41]. In the present review, all the experimental results refer to irradiation with various fs laser beams of samples obtained from intrinsic (resistivity >200 Ω cm), (100) crystalline silicon Czochralski prime wafer (thickness ≈400 μm, typical surface roughness ≤1 nm).

## 3. Grooves Generated by a Gaussian Laser Beam

In this section, we illustrate the surface structures generated by direct fs laser surface structuring by irradiating a standing (static configuration) or a moving silicon sample (dynamic configuration) with a Gaussian laser beam, with emphasis on the supra-wavelength grooves. The fluence spatial profile of a Gaussian laser beam with energy *E_L_* is expressed as $F\left(r\right)=F_{p}exp\left[-2r^{2}/w_{0}^{2}\right]$, where *r* is the radial distance of the point with respect to the beam axis at *r* = 0 and *w_0_* the beam spot radius at 1/*e*^2^ of the peak fluence value $F_{p}=\left(2E_{L}\right)/\left(\pi w_{0}^{2}\right)$.

### 3.1. Static Configuration

**Figure 2:**
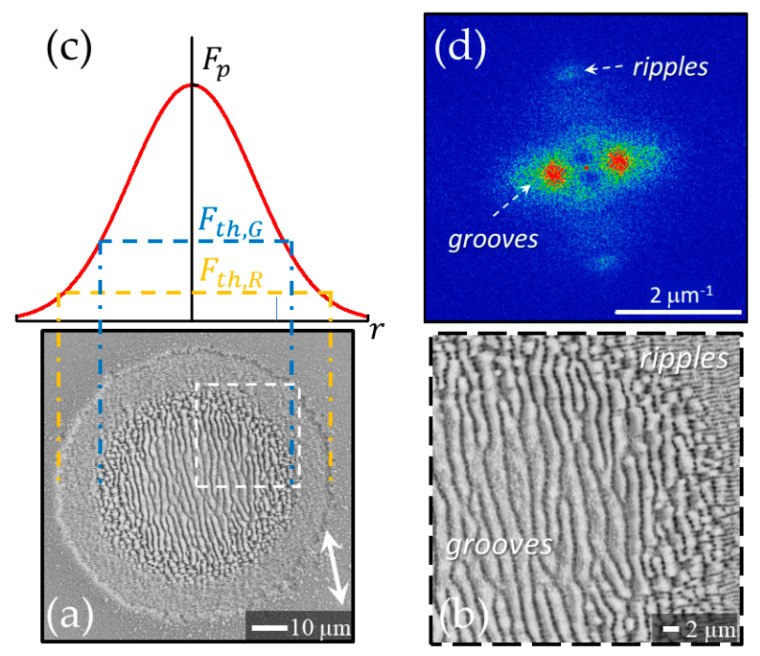
The SEM image in panel (**a**) shows a typical example of surface structures generated on a silicon surface after the irradiation with a Gaussian beam. The double-headed arrow in the SEM image of panel (**a**) shows the direction of the laser beam polarization. Panel (**b**) shows a zoomed view of the area in the white box evidencing the ripples aligned normal to the direction of laser polarization and the micro-grooves oriented along the polarization direction, respectively. Panel (**c**) displays the fluence spatial profile of the Gaussian laser beam and the threshold fluences for the generation of ripples and grooves, respectively. Panel (**d**) shows the 2D-FFT map of the SEM image of panel (**a**) with the characteristic signature of ripples and grooves. The SEM image refers to an irradiation sequence of *N* = 200 laser pulses with ≈35 fs duration at a wavelength of 800 nm for a peak fluence of about 0.7 J/cm^2^.

Figure 2a reports a SEM image that exemplify the typical surface structures generated in static configuration with the laser beam impinging on the target surface at normal incidence. In this configuration, the spatially variant distribution of the fluence typically results in a circular shallow crater decorated with surface structures whose features depend on the location *r* that receives a total fluence dose $\phi_{st}\left(r\right)=NF\left(r\right)$, where *N* is the number of pulses of the laser irradiation sequence. The SEM image shown in panel (a) of Figure 2 refers to a sequence of *N* = 200 laser pulses with ≈35 fs duration at a wavelength of 800 nm and a peak fluence of *F_p_* ≈ 0.7 J/cm^2^ [23,24]. Panel (b) of Figure 2 reports a zoomed view of the area in the white box of panel (a) evidencing the presence of ripples aligned normal to the direction of laser polarization and of grooves oriented along the laser polarization direction, respectively. The ripples decorate a narrow outer annular region, whereas the grooves occupy the main part of the central area of the crater. A very thin intermediate region covered with rudiments of grooves super-imposed over ripples separates the inner and outer areas. This morphology is directly correlated to the radial symmetry of the spatial distribution of the Gaussian beam fluence leading to different features at various location depending on the excitation level induced on the irradiated surface.

The average periods of ripples and grooves estimated from the SEM image are (0.6 ± 0.1) μm and (2.0 ± 0.4) μm, respectively. The typical values of the ablation threshold fluence for silicon registered at 800 nm (pulse width ≈ 35 fs) for irradiation with a sequence of hundred pulses are in the range 0.1–0.2 J/cm^2^ [9,23]; therefore, the used peak fluence is several times larger than the corresponding ablation threshold. As evidenced in Figure 2a, such a condition promotes the generation of the grooves and limits ripples formation to the periphery of the irradiated region. Figure 2c reports a Gaussian fluence profile along the radius of the beam spot in which the thresholds for ripples, *F_th_*_,*R*_, and grooves, *F_th_*_,*G*_, formation are indicated. In the present case, the ripples forms in the region of the laser spot irradiated by local fluence values between ≈0.1 *F_p_* and ≈0.3 *F_p_*, whereas grooves cover all the area at higher fluence from ≈0.3 *F_p_* to the peak value *F_p_* at the center of the beam. Analysis of targets irradiated at higher laser peak fluences indicates that the grooves in the intense part of the beam are progressively destroyed producing columnar structures [23]. Therefore, as for ripples also for the grooves a range of fluences exists for their formation that depends on the specific experimental conditions.

Surface structures characteristics are also analyzed by means of bidimensional fast Fourier transform (2D-FFT) maps of the SEM images [18,20,21]. As an example, panel (d) of Figure 2 reports the 2D-FFT map of the SEM image of panel (a). The 2D-FFT map was elaborated by using Gwyddion software [42]. One can clearly identify couples of characteristic peaks associated to ripples and grooves in the 2D-FFT map aligned along a direction corresponding to the grating formed by the structure disposition in the real space. The features associated to the grooves are more intense due to their prevalence in the SEM image of Figure 2a. The dispersion of the spatial frequencies identified by the vector (*k_x_, k_y_*) is related to the spreading of the values of the spatial period $\Lambda =1/\sqrt{k_{x}^{2}+k_{y}^{2}}$ and of the structures orientation [19,22,24]. The average periods of ripples and grooves estimated from the 2D-FFT image are (0.63 ± 0.05) μm and (1.9 ± 0.5) μm, respectively, in agreement with the values obtained from intensity profile analysis of the SEM image.

The extensions of the part of the shallow crater on which the two different surface structures, ripples, and grooves, develop depend on both the laser peak fluence *F_p_* (or energy *E_L_*) and the number of laser pulses, *N*. At a fixed peak fluence, an increase of both crater size and grooved region occurs with the rise of the pulse number *N* that is associated to a progressive reduction of the fluence thresholds for crater/ripples and grooves formation [9,23,33]. The variation of the fluence threshold *F_th_* with *N* is well described by the incubation behavior expressed by the dependence $F_{th}\left(N\right)=F_{th}\left(1\right)N^{\xi -1}$, where *F_th_*(*N*) and *F_th_*(1) are the fluence thresholds for *N* pulses and single shot irradiation and *ξ* is the incubation factor [9]. The incubation behavior takes into account the progressive changes induced on the sample surface during irradiation with multiple laser pulses. In such processing conditions, feedback mechanisms influence the evolution of the surface morphology varying the effectiveness of laser energy coupling. Such process leads to a gradual lowering of the threshold fluence associated with the formation of specific modifications or structures appearance. As for silicon, Table 1 summarizes the values of the incubation factor registered in the pioneering work of Bonse et al. [9] and in various other investigations carried out on both ripples and grooves by research groups involving the authors of the present review at different laser wavelengths [23,34,43]. For both ripples and grooves values of the incubation factor close to 0.8 are observed at the different laser wavelengths, the scattering of the data being likely due to differences in the experimental conditions, thus suggesting almost no influence of *λ_L_* on *ξ*.

### 3.2. Dynamic Configuration

**Figure 3:**
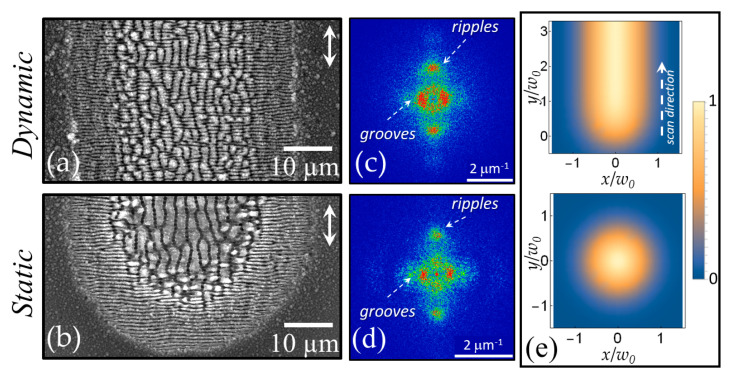
SEM images of the sample surface for (**a**) dynamic and (**b**) static irradiation configurations for surface structuring with laser pulses at 1026 nm with a duration of ≈180 fs. In both cases the laser peak fluence is *F_p_* = 0.4 J/cm^2^. The SEM image of panel (**a**) refers to dynamic irradiation conditions with a scan speed vs. of 0.25 mm/s and a pulse repetition rate *f_L_* = 1 kHz. The lower SEM image shows a portion of the crater generated in static irradiation conditions at the same peak fluence with *N* = 200 laser pulses. The double headed arrows indicate the direction of the laser beam polarization. Panels (**c**) and (**d**) report 2D-FFT maps corresponding to the SEM images obtained for dynamic and static irradiation conditions, respectively. Panel (**e**) displays the normalized fluence dose in the two cases.

Figure 3a reports a SEM image that exemplifies the typical surface structures generated in dynamic configuration with the laser beam impinging on the target surface at normal incidence. For the dynamic conditions, we consider the one-dimensional scanning of the target at a velocity vs. and a laser repetition rate *f_L_* that eventually generates a line on the sample surface. The SEM image shown in panel (a) of Figure 3 refers to a portion of a 1 mm line produced by laser pulses with a duration ≈180 fs and a wavelength of 1026 nm at a peak fluence of *F_p_* ≈0.4 J/cm^2^ for a repetition rate *f_L_* = 1 kHz and a scan speed *V_S_* = 0.25 mm/s [34]. The double headed arrow in Figure 3a indicates the direction of the laser beam polarization that is parallel to the scan direction. The spot size measured on a static target by analyzing the size variation of the generated crater as a function of the pulse energy, at a fixed number of shots, following the procedure introduced by Liu [44] is estimated to be *w*_0_ ≈ 35 μm. Panel (b) of Figure 3 shows a portion of the crater produced on the sample surface for an irradiation sequence of *N* = 200 pulses in static conditions, for comparison. The double headed arrow in Figure 3b shows the direction of the laser beam polarization. The SEM images of Figure 3 display rather similar characteristics of the sample surface with ripples in the peripheral regions at lower fluence and grooves in the area hit by the more intense part of the laser beam. Panels (c) and (d) of Figure 3 reports the 2D-FFT maps of the SEM images of panels (a) and (b), respectively. In both cases, the typical signatures of ripples and grooves can be identified. This observation supports the idea that the mechanisms involved in grooves formation are similar in the two experimental conditions and demonstrate that grooves also can be replicated over large area by target scanning, similarly to ripples. However, in the dynamic case the grooves apparently present a superimposed rippled pattern like that observed in the external part of the line irradiated at lower fluence. This is probably due to the fact that in the dynamic case each position of the line formed on the sample surface is irradiated with a sequence of pulses at a variable value of the fluence due to the raster scanning of the beam, whereas in the static condition the fluence value reaching each location of the irradiated spot is the same for each pulse.

A typical parameter used to quantify the processing conditions in the dynamic configuration is the equivalent number of pulses defined as $N_{eq}=\left(2w_{0}f_{L}\right)/V_{S}$. Actually, for this experimental configuration each location of the irradiated target receives a sequence of pulses at variable fluence: supposing that the target moves along the y-direction, the effective fluence dose received at each position (*x,y*) is expressed as $\phi_{dyn}\left(x,y\right)=F_{p}\overset{N_{tot}}{\underset{k=1}{\sum }}exp\left[-2\left[x^{2}+{\left(y-kV_{s}/f_{L}\right)}^{2}\right]/w_{0}^{2}\right]$, where *N_tot_* is total number of shots used to form a line with a length *L* (i.e., $N_{tot}=f_{L}L/V_{s}$). At a fixed location *y* = y* along the line, the fluence dose received can be expressed as $\phi_{dyn}\left(x,y^{*}\right)=N_{eff}^{*}F_{p}exp\left[-\left(2x^{2}\right)/w_{0}^{2}\right]$, where $N_{eff}^{*}=\overset{N_{tot}}{\underset{k=1}{\sum }}exp\left[-2\left[{\left(y^{*}-kV_{s}/f_{L}\right)}^{2}\right]/w_{0}^{2}\right]$. In Figure 3, the normalized 2D profiles of the fluence dose for static and dynamic configurations are shown in panel (e). The upper image corresponding to the dynamic case evidences that after a distance of few spot sizes *w*_0_, the fluence distribution reaches a stationary behavior. In particular, at fixed position y* along the line the fluence dose along the *x* direction is described by a Gaussian spatial profile with a fluence peak *F_p_* and a corresponding number of pulses $N_{eff}^{*}$ In the present case, $N_{eff}^{*}$ ≈ 180, whereas *N_eq_* = 288, whereas for the static case *N* = 200. In both cases, the topographical features of the SEM images are well associated to the corresponding fluence dose spatial distribution.

The lower number of effective pulses $N_{eff}^{*}$ with respect to the *N* = 200 pulses of the static case explains why the width of the line in panel (a) is slightly smaller than the diameter of the circular spot in panel (b). In addition, it is worth noticing that the way the laser energy is delivered to the target surface remains different in the two configurations: while in the static case the same fluence is delivered by each laser shot, in the dynamic condition a given location y* along the scanning direction experiences a temporally variable irradiation history with a fluence that progressively increases with time, reaches a maximum when the center of the beam spot passes at y* and then gradually reduces [34]. The dynamics of energy supply to the target surface intermingled with the multi-pulse feedback mechanisms eventually leads to the differences in finer morphological characteristics evidenced by the SEM image of Figure 3a,b.

## 4. Grooves Generated by Complex Laser Beams

Recently, direct fs laser surface structuring was also accomplished by exploiting the peculiar properties of complex light beams. Complex (or structured) light beams are characterized by unconventional intensity distribution, polarization state, and phase profile [45,46]. An example of complex light beam is the one carrying orbital angular momentum that is characterized by an annular intensity spatial profile and a variety of polarization states, the radial and azimuthal being the most typical ones. The development of efficient beam converters for the generation of structured laser beams with ultrashort pulse duration has allowed achieving novel spatial distributions of the surface structures [25,31,32,34,35,36,37,38,39,41,44,45,46,47,48,49]. The experimental results shown in this section were achieved by exploiting a q-plate beam converter that generates a vector vortex beam described by a Hyper-Geometric Gaussian mode [50,51,52]. Such a beam mode shows an intensity profile with a main, high intensity annulus followed by a series of secondary, less-intense rings at increasing distances from the beam axis. The main annular component is selected by appropriate beam filtering with a circular aperture leading to a beam profile well approximated by a Laguerre–Gauss mode subclass LG_0,l_ [32,36,43,53,54], that is eventually focused on the target surface.

**Figure 4:**
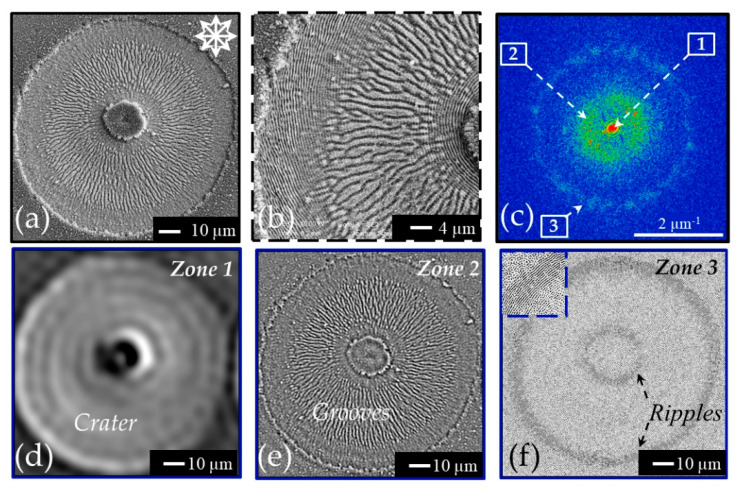
Panel (**a**) reports the SEM image of the sample surface after irradiation with a sequence of *N* = 100 pulses of a radially polarized vector vortex beam at 800 nm with pulse duration of ≈35 fs and energy of *E* ≈ 30 µJ. The zoomed view in panel (**b**) provides a clearer sight of the radial grooves and azimuthal ripples generated on the surface. Panel (**c**) shows a 2D-FFT map obtained from the SEM image of panel (**a**) evidencing three different zones. Panel (**d**–**f**) display the 2D-IFFT of each one of the three different zones separately as indicated in the top-right corner of each image depicting crater, grooves, and ripples, respectively. The dashed box in the upper-left corner of panel (**f**) shows a magnified view of the ripples.

As an example, Figure 4a reports a SEM image of a silicon sample after irradiation with a sequence of *N* = 100 laser pulses of a radially polarized vector vortex beam (wavelength 800 nm, pulse duration ≈35 fs, energy *E* ≈ 30 µJ) [32,33,43,55]. A shallow annular crater is formed that reflects the spatial distribution of the beam intensity. Panel (b) of Figure 4 shows a zoomed view clearly evidencing annular regions with radial grooves and azimuthal ripples surrounding a central assembly of nanoparticles located in the region of the laser beam with almost null intensity. The radial alignment of the grooves and the azimuthal disposition of the ripples, respectively, confirm an orientation parallel and perpendicular to the local polarization of the beam for the two kinds of surface structures. Moreover, the various annular regions decorated with ripples and grooves manifest the existence of fluence intervals favoring the formation of ripples or grooves, similarly to linearly polarized Gaussian beams discussed earlier in Section 3.1.

Panel (c) of Figure 4 shows a 2D-FFT map of the SEM image of panel (a). Interestingly, in this case three different zones can be identified in the 2D-FFT map: a central intense peak (zone 1) and two rings (zones 2 and 3) with different intensities. The radius of the circles of zones 2 and 3 identify vectors in the spatial frequency space with a modulus |*k*| in the ranges (0.5–0.8) μm^−1^ and (1.6–1.9) μm^−1^ (zone 3), respectively; the corresponding spatial periods are in the interval (1.3–2.0) μm and (0.52–0.63) μm and identify zone 2 and 3 as associated to grooves and ripples, respectively. In an attempt to further clarify such an aspect, the 2D inverse FFT (2D-IFFT) of the three zones were elaborated by using the filtering method offered by the Gwyddion software [42]. The 2D-IFFT images are shown in panel (d–f) for the zones 1–3, respectively. From the 2D-IFFT features one can clearly associate the very intense central region of zone 1 to the characteristic of the shallow annular crater, the stronger annulus of zone 2 to the radial grooves located in the central annular region and the weaker external ring of zone 3 to the two external rings covered by azimuthal ripples. The inset in the upper-left corner of panel (f) shows a magnified view of the ripples in the 2D-IFFT map.

**Figure 5:**
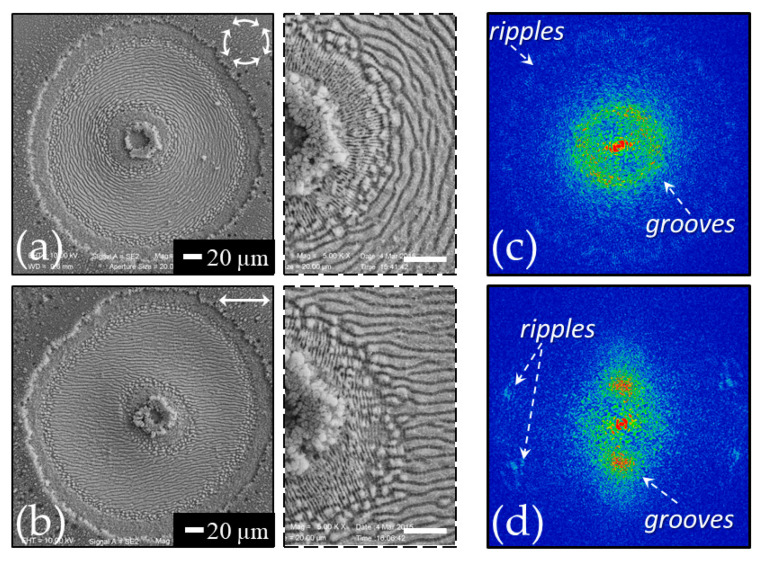
Two examples of craters produced by vector vortex beams with azimuthal (panel **a**) and horizontal (panel **b**) polarization. In both cases the irradiation sequence comprises *N* = 100 laser pulses at an energy *E* ≈ 50 μJ. The lateral insets in the dashed boxes reports zoomed views of the central region evidencing the grooves and ripples. The scale bars in the insets are 5 µm. The right panels (**c**,**d**) show the 2D-FFT maps of the corresponding SEM image.

Figure 5 shows two other examples of the surface structures generated by means of complex laser beams, namely with azimuthal (panel (a)) and linear polarization (panel (b)). Further, in these cases, the zoomed views reported in the dashed box insets clearly illustrate the formation of grooves oriented along the local laser polarization in the most intense part of the laser beam surrounded by ripples on the wings towards the null intensity center and the external area of the annular beam. Panel (c) and (d) of Figure 5 show the 2D-FFT maps corresponding to the SEM images reported in panels (a) and (b), respectively. As for the azimuthal polarization (panels (a) and (c)), the 2D-FFT map shows similar characteristics to those discussed above for the case of radially polarized beam with the three zones associated to the annular crater, the azimuthal grooves, and the radial ripples. The 2D-FFT map of panel (d) evidences the typical intense spots associated to the horizontal grooves seen in panel (b). As for the ripples, four weak features can be ascertained that can be associated to ripples inclined at an angle of about (70–75°) with respect to the horizontal direction of the laser polarization. These inclined ripples can be identified in the dashed box inset of panel (b). The deviation from the expected 90° orientation of the ripples with respect to the horizontal laser polarization is likely due to an imperfect alignment of the optical elements in the beam converter producing the horizontally-polarized annular beam [35]. The examples above reported illustrate a good correspondence of the surface structures orientation with the local laser beam polarization and allow recognizing the analysis of the surface structures as a direct method to visualize the polarization state of high-intensity, complex light beams with a suitable level of spatial resolution and accuracy provided by the periodicity of the surface structures [32,36,38,39,47]. The fact that the grooves are directed along the local polarization of the beam makes them particularly appropriate for such a task, but the ripples can be likewise used for the lower fluence regions of the complex beam. In addition, the relationship of the surface structure with the local values of the pulse fluence provides a qualitative description of the beam spatial fluence distribution.

## 5. Influence of Laser Beam Parameters on Grooves Features

The features of laser-induced surface structures ensue from a quite complex interplay between laser beam properties and material parameters. Ripples have been systematically investigated and, for instance, the dependence of their properties on the various laser characteristics are now well established. For example, ripples period shows a clear, direct dependence of the laser wavelength and a progressive decrease with pulse number associated to the interference mechanisms involving surface scattered waves and multi-pulse feedback effects [10,21,56]. Instead, grooves have been much less investigated and how the various experimental parameters (e.g., laser wavelength, pulse repletion rate, ambient pressure, etc.) influence their morphological features has not been thoroughly studied yet. Hereafter we will briefly illustrate the effects of parameters like number of pulses, laser fluence, and wavelength on the grooves period.

**Figure 6:**
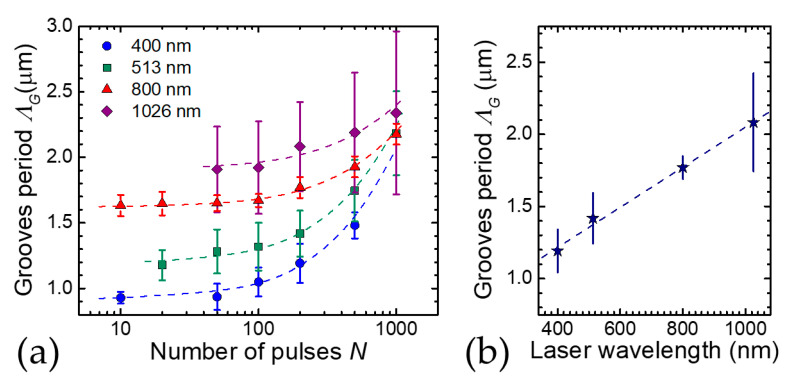
(**a**) Variation of the grooves period *Λ_G_* as a function of the number of pulses *N* for four different laser wavelengths. The data refer to laser surface texturing with Gaussian beams at different laser wavelengths: circles—400 nm (≈100 fs, *F_p_* = 0.27 J/cm^2^) [31]; squares—513 nm (≈180 fs, *F_p_* = 0.4 J/cm^2^) [34]; triangles—800 nm (≈35 fs, *F_p_* = 0.5 J/cm^2^) [33]; diamond—1026 nm (≈180 fs, *F_p_* = 0.4 J/cm^2^) [34]. The dashed lines represent linear fits to the experimental data (note that data are reported on a linear-log plot). (**b**) Grooves period vs. laser wavelength at *N* = 200 laser pulses.

Figure 6a summarizes experimental results on the variation of the grooves period, *Λ_G_*, as a function of the number of pulses, *N*, obtained by irradiating a crystalline silicon target with laser beams at four different laser wavelengths. The period of the grooves slightly varies with location, therefore in Figure 6a average values are shown as symbols and the variability is expressed by the error bars. The data have been collected from References [31,33,34]. The corresponding experimental conditions are as follows: for 400 nm—pulse duration *τ_L_* ≈ 100 fs, peak fluence *F_p_* = 0.27 J/cm^2^ [30]; for 513 nm—*τ_L_* ≈ 180 fs, *F_p_* = 0.4 J/cm^2^ [33]; for 800 nm: *τ_L_* ≈ 35 fs, *F_p_* = 0.5 J/cm^2^ [32]; and for 1026 nm *τ_L_* ≈ 180 fs, *F_p_* = 0.4 J/cm^2^ [33]. Figure 6a clearly shows that, at a fixed peak fluence, *Λ_G_* progressively rises with *N*. In particular, the increasing trend of *Λ_G_* on *N* seems to be well described by a linear dependence as shown by the dashed lines in Figure 6a (note that data are reported on a linear-log plot). Analyses of the grooves period, at a fixed number of pulses, also show a progressive, approximately linear rise on the laser beam peak fluence (or pulse energy) [33,34]. Similar tendencies have been also observed for grooves generated by complex light beams [32,33]. These trends are very much different from those observed for ripples, thus suggesting that other mechanisms might be into play for the generation of grooves. Nevertheless, Figure 6a also evidences a clear dependence of the value of the grooves period on the laser wavelength. In this respect, it is worth noticing that the wavelength influences the energy coupling to the target due to the variation of the photon energy and material absorption efficiency. As for the wavelengths considered here, the registered values of the fluence threshold for material ablation at *N* = 100 pulses have been estimated to be ≈40 mJ/cm^2^ at 400 nm and 513 nm and ≈90 to 100 mJ/cm^2^ at 800 nm and 1026 nm. Therefore, the data of Figure 6a refer in all cases to experimental conditions characterized by a fluence well above the ablation threshold and in which grooves formation is favored. To show in a clearer way how the laser wavelength affects the grooves period, Figure 6b reports the values of *Λ_G_* on the laser wavelength for *N* = 200 laser pulses taken from Figure 6a. The observed tendency suggests an evident correlation between laser wavelength and grooves period, supporting the idea that coherent feedback mechanisms related to the laser wavelength observed for the ripples may also be involved in the mechanisms underlying the formation of grooves.

**Figure 7:**
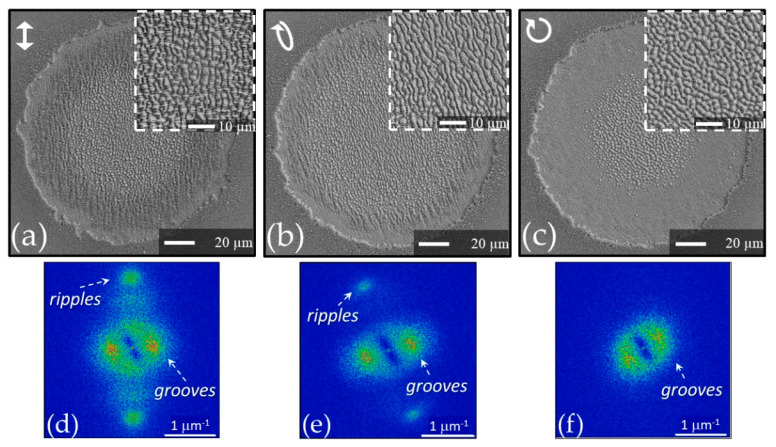
SEM images of the craters produced with a Gaussian beam at wavelength of 1055 nm and pulse duration of ≈900 fs for three different states of polarization: (**a**) linear, (**b**) elliptical, and (**c**) circular. In each condition, the surface is irradiated with *N* = 200 laser pulses at an energy of *E* ≈ 150 μJ. In each panel (**a**–**c**), the upper-right inset shows a zoomed view of the central region of the SEM image. Panels (**d**–**f**) report the 2D-FFT maps obtained from the corresponding upper panels (**a**–**c**). The graph in the upper-left corner of panels (**a**–**c**) represents the beam polarization.

Another intriguing aspect deserving further analysis is the influence of the laser beam polarization. In Section 4, grooves formed by using complex light beams with spatially varying polarization were illustrated. Here we address the changes induced on the grooves by turning the polarization of a Gaussian laser beam from linear to elliptical and eventually to circular, in the same experimental conditions. Figure 7 reports three SEM images of the shallow craters produced by irradiation with a Gaussian beam at 1055 nm with laser pulses duration of ≈900 fs and an energy ≈150 μJ [57,58,59]. The laser spot size is *w_0_* ≈ 130 μm and the peak fluence is *F_p_* ≈ 0.57 J/cm^2^. For each condition, the surface is irradiated with *N* = 200 laser pulses. In each one of the panels (a–c), the upper-right inset shows a zoomed view of the central region of the main SEM image. Panels (d–f) report the 2D-FFT maps obtained from the corresponding upper panels (a–c). The graph in the upper-left corner of panels (a–c) schematically shows the beam polarization. In all the three cases a crater with a radius of about 70 μm is generated, but the features of the structures decorating its surface vary with the laser beam polarization. For linear polarization, the SEM image of panel (a) shows a central region with a radius of ≈ 50 μm covered by grooves and surrounded by a rippled ring. As shown in the corresponding 2D-FFT map in panel (d), the ripples and grooves are oriented along the directions perpendicular and parallel to the to the laser beam polarization, respectively. The period of ripples can be estimated as (0.74 ± 0.06) μm, whereas grooves period is (2.5 ± 0.7) μm. Turning to the elliptical polarization, panels (b) and (e) evidence the formation of ripples and grooves oriented at about 30° counterclockwise with respect to linear polarization, i.e., along the perpendicular and the parallel to the direction of the major axis of the polarization ellipse, respectively. In this case also, the ripples cover an external ring encircling a central grooved area with a radius of ≈ 50 μm. The ripples and grooves periods are estimated to be (0.75 ± 0.04) μm (2.2 ± 0.6) μm, in agreement with the previous case. Instead, for circular polarization, in panels (c) and (f), there is no signature of ripples, but features with a rather different morphology are observed in a central part of the crater covering a smaller area with a radius of ≈35 μm showing a spatial periodicity extending over a larger range going from ≈1.7 μm to ≈4.4 μm with an average of ≈3 μm. Moreover, the 2D-FFT map suggests a larger angular spread, even if a kind of preferential orientation seems to be still present possibly due to an imperfect generation of a circularly polarized beam. These observations indicate that, differently from ripples, circular polarization induces topographical features with a lower degree of spatial order and over a reduced crater area; that is, suggestive of a larger threshold fluence for their formation. Consequently, one can speculate that the presence of ripples somehow influences the grooves formation and regularity. Further observation for the circularly polarized case will be illustrated later in Section 6 addressing differences between processing in air and vacuum.

## 6. Experimental Conditions Hindering Grooves Formation

Interestingly, there are some experimental conditions that seem to prevent the formation of grooves besides the disappearance occurring at high laser peak fluences and large number of pulses leading to generation of columnar structures [23,34]. Here we specifically refer to experimental observations related to both laser surface texturing in high vacuum [57,58], at high repetition rate in air [60,61] and with a circularly polarized beam.

**Figure 8:**
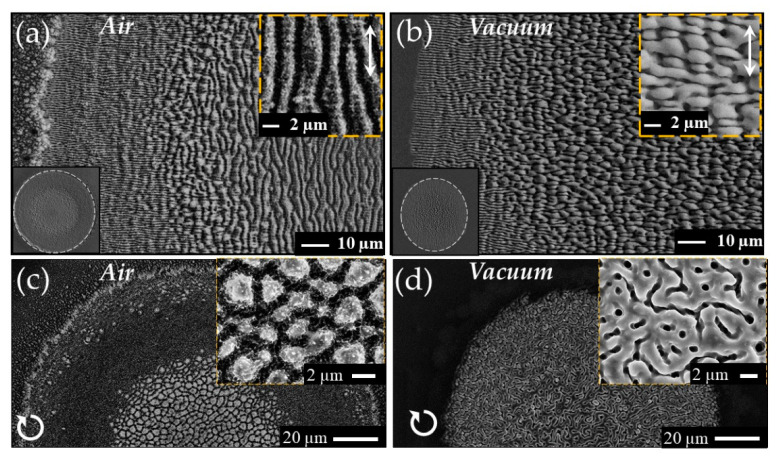
Examples of SEM images of surface of a silicon target irradiated with a sequence of *N* = 300 laser pulses at a peak fluence of ≈0.65 J/cm^2^ in (**a**) air and (**b**) high vacuum. Panels (**a**,**b**) refer to a surface processed with linear polarization, whereas panels (**c**,**d**) are for a circular polarization, as shown by the white arrows schematically indicating the polarization state. Moreover, panels (**a**,**c**) are for air and (**b**,**d**) for vacuum, respectively. The insets in the upper right corner of each panel shows views of the surface registered at higher magnification. The insets in lower left corners of panels (**a**,**b**) reports SEM images evidencing the change in the shape of the shallow crater from circular to elliptic in air and vacuum, respectively.

As an example, Figure 8 reports SEM images of silicon samples obtained in air at atmospheric pressure (panels (a) and (c)) and in vacuum at a residual background pressure of 10^−4^ mbar (panels (b) and (d)) with a sequence of *N* = 300 laser pulses. The laser source used in the experiment provided pulses at a wavelength of 1055 nm with a duration of ≈900 fs that were focused on the target at normal incidence achieving a beam spot with a size of about 130 μm. Considering first the case of irradiation with linear polarization in air (panel (a)), the surface structures show the typical features described above: grooves parallel to the laser polarization in the central region surrounded by an external annulus covered by ripples perpendicular to the laser polarization. In vacuum (panel (b)), instead, the external border of the shallow crater is still formed by a rippled ring, but the internal part irradiated by higher laser fluence, typically covered by grooves in air, shows quite different morphological features. In fact, the surface presents a multicellular pattern formed by elements preferentially elongated in direction perpendicular to laser polarization and characterized by a length ranging from few to tens of μm. The observed morphology seems to suggest that these structures may form by a merging of two or more adjacent ripples as a consequence of the higher fluence and the multi-pulse irradiation.

Strikingly, the experimental conditions also influence the size and shape of the shallow crater formed on the target as illustrated by the SEM images reported in the lower-left insets of panels (a) and (b) whose edges are evidenced by dashed white lines. In air, the crater presents the typical circular shape associated to the Gaussian fluence profile of the laser beam (inset of panel (a)), whereas in vacuum a slightly smaller, elliptic crater with major axis directed along the laser pulse polarization is formed (inset of panel (b)). The elliptic shape of the crater in vacuum was rationalized by considering the effect of surface scattered waves along the polarization direction favored by the ripples and surface roughness decorating the surface during the multi-pulse irradiation sequence. This aspect was further supported by analyzing the effects of the variation of the laser beam polarization in vacuum: by changing the direction of the linear polarization a rotating elliptical spot with major axis directed along the polarization was observed, while using elliptical and circular polarizations a progressive tendency towards the formation of more circular craters occurred [58].

Panels (c) and (d) of Figure 8 report examples of the SEM images of a sample processed with a circularly polarized laser beam in air and vacuum, respectively. Two remarks are in order here: (i) in both cases no ripples are generated as expected for circular polarization; (ii) the central region of the crater is covered with structures showing rather different morphology. In air the surface mainly presents columnar structures with a typical size of 1–2 μm, whereas in vacuum it is characterized by a complex pattern formed by stripes with lengths of few μm and a characteristic valley width of about 100 nm. An important difference between the two cases is the large presence of nanoparticles decorating the outer edge of the crater in air that are almost absent in vacuum. This nanoparticles are mainly due to back-deposition of the ablation nanoparticulate generated during fs laser ablation at high fluence that can be effectively confined and pushed back towards the target surface in air while expanding and flying away in vacuum [62,63]. Interestingly, the different morphology of the target surface in the two cases, i.e., a rougher and grainer surface texture in air than in vacuum as a consequence of nanoparticles decoration, modifies the surface optical absorption [64,65]. The larger absorption of the rougher surface that progressively forms under laser irradiation in air leads to a lower multi-pulse ablation threshold with respect to vacuum [59].

**Figure 9:**
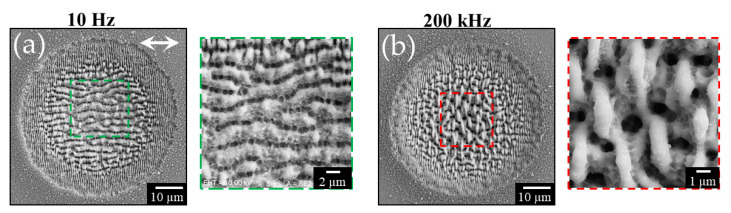
Examples of SEM images of a silicon target irradiated with a sequence of *N* = 200 laser pulses at a peak fluence of ≈0.5 J/cm^2^ for repetition rates of (**a**) 10 Hz and (**b**) 200 kHz. The white arrow in panel (**a**) schematically indicates the direction of the linear polarization. The insets on the right of each SEM images reports views of the area in the dashed, colored box registered at higher magnification.

Other experimental conditions leading to the disappearance of the grooves have been observed during experiments on fs laser surface structuring of silicon at repetition rates variable in the range 10 Hz–200 kHz [60,61]. Exemplificative results are shown in Figure 9. Supra-wavelength grooves are formed in the central region of the shallow crater at low repetition rate, as shown for example in panel (a) of Figure 9 for *N* = 200 laser pulses at a peak fluence of ≈0.5 J/cm^2^ for a repetition rate of 10 Hz. For repetition rates larger than about 10 kHz, the experimental findings showed both a reduction of the crater size and the formation of a network of micro-sized globular, crochet-like structures in the central region of the crater. As an example, panel (b) of Figure 9 reports the SEM image of the sample irradiated at 200 kHz with the same sequence of *N* = 200 laser pulses. Both these experimental observations were ascribed to a shielding effect of the sample surface occurring at high repetition rate due to the nanoparticulate plume generated during the ablation process ensuing laser irradiation. This finding, in turn, suggests that the cloud of ablated material formed above the sample surface might influence the mechanisms involved in grooves formation. At high repetition rate, the train of laser pulses interacting with the plume of ablated nanoparticles can effectively reduce their back-deposition, besides limiting the energy coupling to the target. Hence, the experimental observations illustrated above for fs laser surface texturing in vacuum and at high repetition rate seem to support a scenario in which also the presence of the nanoparticles formed during the ablation process, accompanying the irradiation at the higher fluences that favor the generation of grooves, may play a role in the complex mechanisms leading to grooves formation [23,24]. However, how such an effect takes place deserves still further investigations to fully comprehend and elucidate its specific role.

## 7. Possible Mechanisms Involved in Grooves Formation

Currently, fs LIPSS formation is namely addressed by two approaches related to mechanisms involved both in the laser energy deposition into the solid target and reorganization and redistribution of near-surface matter. The various advancements of the theoretical description of the LIPSS formation process and the different physical mechanisms considered have been recently reviewed by Bonse and Gräf [19] and Bonse [18]. However, most of the models primarily deal with low spatial frequency ripples and a complete theory for the high spatial frequency ripples is still under development. The processes leading to grooves formation have been investigated even less, therefore some of the mechanisms involved in their generation have been addressed only recently and a comprehensive understanding has not been achieved yet.

The irradiation of the target surface by fs laser pulses is described by the Two-Temperature-Model (TTM), with energy coupling to the electronic subsystem and subsequent transfer to the lattice over a timescale longer than the pulse duration [66,67]. For intrinsic semiconductors, of which silicon is often considered as a case study, the amount of free electrons in the conduction band at room temperature is negligible, therefore electrons excitation through single or multi-photon absorption from the valence to the conduction band should occur depending on wavelength and intensity of the laser pulses. The consequent change of the electron number density in the conduction band generates a high carrier density, on a timescale shorter than the electron–phonon relaxation time, and modifies the material dielectric permittivity *ε* transiently turning it into a metallic state with the possibility of Surface Plasmon Polaritons (SPP) excitation (*Re*{*ε*} < −1) [21,56,66,67,68,69]. On the surface of the material in this excited state SPPs or scattered surface wave (SSW) induced by defects are launched and interfere with the incident laser pulse producing a modulation of the energy coupled to the electron subsystem. The ensuing transfer of energy to the lattice leads to the imprinting of an absorbed energy landscape characterized by a spatial modulation with a period slightly smaller than the laser wavelength. This scenario is the most accepted explanation for the formation of the periodic, subwavelength ripples and explains the dependence of their spatial features (e.g., period and orientation) on polarization and wavelength of the laser pulses. Interestingly, simulation of the spatial distributions of deposited laser energy on a silicon target surface evidences the formation of another modulation, besides that associated to the ripples, in a region characterized by a higher excitation level [24,30]. Strikingly, this secondary modulation is arranged along a direction perpendicular to that corresponding to the ripples. Therefore, at higher fluence, a (quasi-)periodic grating with larger stripes parallel to the laser polarization with a spatial scale comparable to that experimentally observed for the grooves period accompanies the one related to ripples [24,30,32]. This observation seems to support a scenario in which the electromagnetic mechanism that leads to redistribution of laser pulse energy might also contribute to grooves formation. Such an aspect is clearly illustrated by the experimental findings obtained by irradiation with fs complex beams with spatially varying polarization state in Section 4. Since the electromagnetic processes are directly related to the laser wavelength [70], such an interpretation seems in agreement with the wavelength variation of the grooves period observed in the experiments discussed earlier in Section 5 (see Figure 6).

A more complete model of surface structures induced by fs laser pulses has been developed by Tsibidis et al. complementing TTM and electromagnetic process with hydrodynamics [25,30]. This approach has allowed to get more direct evidence of grooves formation and particularly of the dependence of their period on the experimental parameters [30]. The model clearly addresses a variation of the grooves period, *Λ_G_*, on the laser pulse number, *N*, predicting a progressive increase of *Λ_G_* on *N*, in agreement with the experimental observations illustrated in Figure 6a [31,32,34]. The results of these simulations suggest that hydrodynamics and melt flow dynamics induced at high excitation level can contribute to the generation of the grooves. In particular, at high excitation level, which can be associated to a relatively high number of laser pulses or larger fluences, when the grooves are well-developed there is an enhancement of the temperature gradient driving the melt flow primarily in a direction normal to the laser polarization; that is, in the direction of grooves. Recently, a direct comparison between the predictions of this modeling approach and experimental findings on the variation of *Λ_G_* on *N* at two laser wavelengths showed a fairly good agreement [34].

Another simulation approach used to investigate LIPSS formation is based on Finite Difference Time Domain (FDTD) methods [70,71,72,73,74,75]. In this context, Skolki et al. analyzed the spatial distribution of the absorbed energy for a rough silicon surface irradiated by fs laser pulses [71,72]. The rough surface was realized by considering topographic defects randomly distributed over a flat surface. These simulations evidenced the possible generation of different surface structures and a classification was defined by using the characteristic features in the Fourier space. Among others, the analysis showed the formation of surface structures parallel to the laser polarization with a characteristic period larger than the wavelength dubbed “type g” structures due to scattering mechanisms [19,71]. These supra-wavelength structures are associated to the grooves observed in the experiments on semiconductors and metals and their pattern in the Fourier space is similar to that observed for grooves in the 2D-FFT maps reported in the previous sections. In other FDTD studies, Zhang et al. simulated the multi-pulse irradiation of a rough surface of W and Si observing the formation of a spatial pattern of energy absorption coherent with both low and high spatial frequency ripples as well as grooves [70]. These FTDT studies suggest an influence of the electromagnetic processes of the origin of grooves related to the progressive evolution of the topography of a rough surface driven by feedback effects associated with the fs multi-pulse irradiation. Such effects were induced by a randomly distributed rough surface with nanoparticles characterized by heights and widths larger than about 100–200 nm in the simulations carried out by Zhang et al. [70].

The results of the FTDT methods support a scenario in which the grooves can be induced by the electromagnetic effects related to scattering from surface defects, like nanoparticles, leading to a redistribution of the energy coupled to the target that depend on feedback mechanisms associated to multi-pulse irradiation conditions. These processes take place on the short timescale of the pulse duration. The successive evolution of surface topography and grooves characteristics involves, on a longer timescale, mechanisms related to melt flow dynamics and hydrothermal effects, as clarified by the approach of Tsibidis et al. in which hydrodynamics is coupled to the electromagnetic processes. In addition to addressing the formation of grooves during fs laser irradiation of silicon, such a scenario seems to offer a way to rationalize the observed dependence of the grooves period on the laser wavelength (see Section 5) and the possible role of nanoparticles (see Section 6). In such a picture, the hampering of grooves generation in some experimental conditions illustrated in Section 6 might be associated to the limitation of an effective nanoparticulate coverage of the irradiated surface. However, these last aspects still deserve further theoretical and experimental investigations to fully clarify the complex interplay between the various physical mechanisms involved in the process and completely explain the different topographic features observed in vacuum and at high repetition rate.

## 8. Summary

Formation of fs LIPSS has been established as a remarkable way to create a variety of surface textures with a wide range of applications. In addition to the several LIPSS with a subwavelength period, other supra-wavelength surface structures indicated as grooves form during fs laser irradiation. Grooves typically form at large fluence and/or high number of laser pulses. In this review, the current results of various recent experimental investigations on grooves formation on crystalline silicon irradiated by fs laser pulses carried out by the authors has been reviewed. The dependence of the grooves properties on experimental parameters like laser polarization, wavelength, fluence, repetition rate as well as number of laser pulses, irradiation conditions (e.g., static or moving target, vacuum, or atmospheric pressure environment) has been illustrated. The experimental findings clearly evidence the possibility to tailor features like orientation and period of the grooves using complex light beams or laser wavelength, similarly to the behavior of subwavelength low spatial frequency ripples. Moreover, the dependency on the number of laser pulses, *N,* and peak fluence, *F_p_*, shows a trend opposite to ripples with grooves period progressively rising with *N* or *F_p_*. Finally, some experimental conditions that hamper grooves formation have been identified as large values of the laser fluence, vacuum processing, circular polarization, and irradiation at high repetition rate in air. All the observed features have been rationalized in the frame of current models of LIPSS formation involving a complex interplay between the spatial redistribution of the energy coupled to the target, due to scattered waves generated on a rough surface, hydrothermal and melt flow dynamics of the excited material and feedback effects associated to multi-pulse laser irradiation. Finally, the observation that grooves can be effectively extended over a large area, by laser or target scanning, might trigger interest for applications of such structures in imparting functionalities to materials surfaces, similarly to what has been realized for ripples in the last decades. In addition to their use in the direct visualization of the polarization state of complex light beams, applications of grooves have not been addressed yet and stimulating research activities might be carried out in the years ahead.

## Figures and Tables

**Table 1:** Values of the incubation factor *ξ* registered for laser surface structuring of silicon at different laser wavelengths *λ_L_* both for ripples and grooves.

*λ*_L_ (nm)	Incubation Factor *ξ*		Reference
	*Ripples*	*Grooves*	
513	0.87 ± 0.02	0.82 ± 0.06	[34]
800	≈0.84	-	[9]
800	0.76 ± 0.04	0.82 ± 0.03	[23]
800	0.81 ± 0.05	-	[43]
1026	0.78 ± 0.04	0.77 ± 0.03	[34]

## Data Availability

Data sharing not applicable. No new data were created or analyzed in this study. Data sharing is not applicable to this article.
